# Short-lived reactive components substantially contribute to particulate matter oxidative potential

**DOI:** 10.1126/sciadv.adp8100

**Published:** 2025-03-19

**Authors:** Steven J. Campbell, Battist Utinger, Alexandre Barth, Zaira Leni, Zhi-Hui Zhang, Julian Resch, Kangwei Li, Sarah S. Steimer, Catherine Banach, Benjamin Gfeller, Francis P. H. Wragg, Joe Westwood, Kate Wolfer, Nicolas Bukowiecki, Mika Ihalainen, Pasi Yli-Pirilä, Markus Somero, Miika Kortelainen, Juho Louhisalmi, Martin Sklorz, Hendryk Czech, Sebastiano di Bucchianico, Thorsten Streibel, Mathilde N. Delaval, Christopher Ruger, Nathalie Baumlin, Matthias Salathe, Zheng Fang, Michal Pardo, Sara D’Aronco, Chiara Giorio, Zongbo Shi, Roy M. Harrison, David C. Green, Frank J. Kelly, Yinon Rudich, Suzanne E. Paulson, Olli Sippula, Ralf Zimmermann, Marianne Geiser, Markus Kalberer

**Affiliations:** ^1^ MRC Centre for Environment and Health, Environmental Research Group, Imperial College London, 86 Wood Lane, London W12 0BZ, UK.; ^2^ Department of Environmental Sciences, University of Basel, Klingelbergstrasse 27, 4056 Basel, Switzerland.; ^3^ Department of Atmospheric and Oceanic Sciences, University of California at Los Angeles, 405 Hilgard Ave, Portola Plaza, Los Angeles, CA 90095-1565, USA.; ^4^ Institute of Anatomy, University of Bern, 3012 Bern, Switzerland.; ^5^ Department of Environmental Science, Stockholm University, 106 91 Stockholm, Sweden.; ^6^ Yusuf Hamied Department of Chemistry, University of Cambridge, Lensfield Road, Cambridge, CB2 1EW, UK.; ^7^ Institute of Molecular Systems Biology, ETH Zürich, Zürich, Switzerland.; ^8^ Department of Environmental and Biological Sciences, University of Eastern Finland, P.O. Box 1627, 70211 Kuopio, Finland.; ^9^ Institute of Chemistry, University of Rostock, Albert-Einstein Str. 27, 18051 Rostock, Germany.; ^10^ Comprehensive Molecular Analytics (CMA), Helmholtz Zentrum München, 85764 Neuherberg, Germany.; ^11^ Department of Internal Medicine, University of Kansas Medical Centre, Kansas City, KS 66160, USA.; ^12^ Department of Earth and Planetary Sciences, Faculty of Chemistry, Weizmann Institute of Science, Rehovot, Israel.; ^13^ Departimento di Scienze Chimiche, Università degli Studi di Padova, via Marzolo 1, Padova 35131, Italy.; ^14^ Division of Environmental Health and Risk Management, School of Geography, Earth and Environmental Sciences, University of Birmingham, Birmingham B1 52TT, UK.; ^15^ NIHR HPRU in Environmental Exposures and Health, Imperial College London, London, UK.; ^16^ Department of Chemistry, University of Eastern Finland, P.O. Box 111, 80101 Joensuu, Finland.

## Abstract

Exposure to airborne particulate matter (PM) has been attributed to millions of deaths annually. However, the PM components responsible for observed health effects remain unclear. Oxidative potential (OP) has gained increasing attention as a key property that may explain PM toxicity. Using online measurement methods that impinge particles for OP quantification within seconds, we reveal that 60 to 99% of reactive oxygen species (ROS) and OP in secondary organic aerosol and combustion-generated PM have a lifetime of minutes to hours and that the ROS activity of ambient PM decays substantially before offline analysis. This implies that current offline measurement methods substantially underestimate the true OP of PM. We demonstrate that short-lived OP components activate different toxicity pathways upon direct deposition onto reconstituted human bronchial epithelia. Therefore, we suggest that future air pollution and health studies should include online OP quantification, allowing more accurate assessments of links between OP and health effects.

## Teaser

Reactive chemical components in ambient particulate matter are key drivers of their oxidative potential.

## INTRODUCTION

Exposure to ambient particulate matter (PM) was estimated to be the seventh leading risk factor for all-age mortality in 2019 (*1*) and is associated with a wide range of adverse health outcomes (*2*–*5*). The World Health Organization recently reduced guideline annual exposure limits for PM with an aerodynamic diameter <2.5 μm (PM_2.5_) from 10 to 5 μg m^−3^. Considering this recent update, 99% of the world’s population live in locations that exceed World Health Organization guideline limits for this pollutant. Reducing PM_2.5_ mass concentrations below these guideline thresholds to limit their impacts on human health is challenging. Therefore, future air pollution mitigation strategies will require toxicity-specific PM metrics to identify the most harmful particle components, which may originate from a wide range of anthropogenic and natural sources. PM is composed of tens of thousands of mostly organic, highly oxidized and functionalized compounds but also contains inorganic salts and metals (*6*–*8*). Organic aerosols generated in the atmosphere (rather than emitted directly from the source) through chemical and physical gas-to-particle conversion mechanisms, typically involving oxidation reactions, are defined as secondary organic aerosol (SOA). In PM_2.5_, SOA is often a dominant mass fraction, containing substantial amounts of peroxides and other redox-active components such as quinones, which are chemically reactive and relatively short lived (*6*, *8*, *9*).

A growing body of evidence suggests that oxidative stress, an imbalance of the oxidant-to-antioxidant ratio favoring the former, which can be promoted by PM upon exposure to the lung, is a fundamental mechanism of PM toxicity (*10*–*12*). PM-mediated oxidative stress can promote cell damage, increase inflammatory and immune responses, and exacerbate symptoms in preexisting pulmonary diseases such as asthma and chronic obstructive pulmonary disease, as well as in cardiovascular diseases (*13*, *14*). Reactive oxygen species (ROS), typically referring to the sum of the hydroxyl radical (^.^OH), superoxide radical (O_2_^·−^), and hydrogen peroxide (H_2_O_2_), in some cases including organic peroxides (ROOH) and organic radicals, contribute to PM-induced oxidative stress, as do redox-active transition metals (*13*). The capability of particles to produce ROS and promote redox chemistry with simultaneous reduction of antioxidant concentrations in the lung is defined as oxidative potential (OP) (*10*). PM components including anthropogenic SOA, biomass burning organic aerosol, and redox-active transition metals have been identified as especially OP-active PM components by previous studies (*15*–*17*). While OP has emerged as a metric that may provide a crucial link between particle composition, toxicity, and adverse human health effects, standardized measurement methodologies have yet to be developed despite increasing and widespread measurement of OP by research groups globally (*18*). In addition, uncertainty remains regarding the direct attribution of OP to adverse health outcomes (*18*). Positive associations have been made between acellular OP and health end points such as increased asthma/wheeze (*19*) and fractional exhaled nitric oxide (*20*), in some cases with stronger associations than PM mass concentration alone (*18*). Despite this, multiple studies using modeling and measurement approaches have also found null associations between OP and a range of respiratory and cardiovascular end points (*18*). These conflicting results, and thus the lack of a robust link between OP and adverse health effects, may be partially due to the wide range of OP measurement methods applied. Established methods for quantifying OP rely on offline analysis, where filter samples are collected and typically stored for hours to months before analysis. However, a substantial fraction of the components contributing to OP, such as radicals and organic peroxides, are highly reactive and short lived (*18*, *21*, *22*). Therefore, offline analysis of filter samples likely underestimates the true OP and particle-bound ROS burden of PM because of decomposition of reactive species (*22*–*26*). To address this analytical-chemical challenge, we have developed online measurement methods for OP and ROS quantification. They provide robust, rapid quantification of particle OP and ROS using a direct-to-reagent sampling approach, impinging particles into reagents in a flow-through configuration within seconds in situ. In addition, they provide highly time-resolved data (5 to 10 min), while typical offline methods have a time resolution of ~24 hours (*25*, *27*–*29*). The application of online approaches substantially improves our capacity to quantify OP more robustlywith improved temporal resolution compared to previous studies using offline approaches. However, at present, there is no comprehensive assessment of the stability of particle-bound ROS and OP.

Here, we use two online instruments using different acellular chemical assays, measuring the sum of short-lived and stable ROS and OP. The sum of stable and short-lived ROS and OP as quantified with our online instruments is defined here as total ROS (ROS_T_) and total OP (OP_T_) (*25*, *29*, *30*). We reveal that established filter-based techniques underestimate ROS_T_ and OP_T_ levels by up to a factor of 100. Large variability in the fraction of short-lived ROS and OP of PM from different emission sources and sample locations makes it challenging to extrapolate OP_T_ and ROS_T_ from offline measurements. We also show that online deposition of particles onto reconstituted human bronchial epithelia cultured at the air-liquid interface (ALI) and offline deposition of aqueous particle extracts onto the same cells trigger substantially different biological responses, which may be attributed to short-lived OP components. Both aspects highlight that robust quantification of OP using online direct-to-reagent sampling methods is essential to identify the link between particle OP and toxicity to facilitate more effective air pollution reduction measures in the future.

## RESULTS

### OP and ROS in particles are short lived

**Fig. 1.**
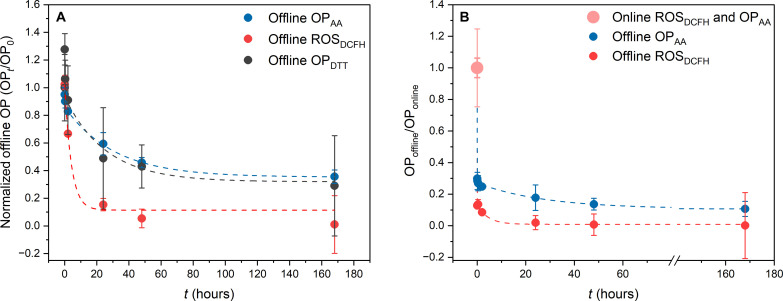
ROS and OP in biogenic SOA decay within hours. (**A**) Offline analysis (OP*_t_*/OP_0_) showing filter-based ROS_DCFH_ (red), OP_DTT_ (gray), and OP_AA_ (blue) decay in biogenic β-pinene SOA for all three main metrics used to quantify ROS and OP. SOA particles were collected on filters for 100 s before immediate analysis. The same filters were then analyzed periodically while being stored for up to a week. OP_0_ is the offline OP measurement immediately after extraction and OP*_t_* at later time points. Data between 0- and 20-min decay times are shown and discussed in fig. S2. The very short particle collection and processing times add to measurement uncertainty, and the OP_DTT_ data point at 5 min is 30% higher than the data point at 2 min, which is within experimental uncertainty observed over three repeats. (**B**) Offline ROS_DCFH_ and OP_AA_ decay curves [same data as in (A)] combined with online quantification (pink data point) normalized to online data (OP_offline_/OP_online_). The thick pink error bar represents online ROS_DCFH_ uncertainty, and the thin error bar represents online OP_AA_ uncertainty. Analyses show that only 10% of OP_T_ and 1% of ROS_T_ are stable for longer than a week. OP_AA_ data were adapted from Utinger *et al.* (*25*). Fits in (B) assume two OP components in SOA with different reaction rate constants decaying with first-order kinetics and one stable OP component: a rapid decay of ~85% of ROS_T_ and ~75% of OP_T_, with half-lives of 1.1 and 1.4 min, respectively, followed by a slower second component (~14% for ROS_T_ and ~15% for OP_T_), decaying with half-lives of 3.6 and 37 hours, respectively, leaving stable components (~1% for ROS_T_ and 10% for OP_T_) with a half-life of >1 week.

We characterized the lifetime of OP and ROS of biogenic SOA particles, generated in the laboratory and collected on Teflon filters. Offline OP and ROS were quantified using three widely used acellular methods: 2,7-dichlorofluorosecein (DCFH; ROS_DCFH_), dithiothreitol (DTT; OP_DTT_), and ascorbic acid (AA; OP_AA_). Time-resolved decay of ROS_DCFH_, OP_AA_, and OP_DTT_ is presented in Fig. 1A. We demonstrate that across all offline assays, OP and ROS decay with a half-life of a few hours to about 1.5 days, which is likely due to the decomposition of labile components such as peroxides (*23*, *30*, *31*) (see subsequent discussion). Only a small fraction is stable for more than 7 days.

Only when the same particles were measured with online instruments (*25*, *29*, *30*) (where particles were directly impinged and quantified with AA or DCFH assays within seconds, therefore capturing the highly reactive fraction of OP_T_ and ROS_T_ that offline measurements used in Fig. 1A miss), the large fraction of short-lived ROS- and OP-active components that contribute to OP_T_ and ROS_T_ could be quantified (Fig. 1B). Online ROS_DCFH_ and OP_AA_ measurements were a factor of 5 and 3 larger, respectively, than the fastest offline analysis, where the delay between particle collection and analysis was only 2 min. After 2 days, ROS_DCFH_ and OP_AA_ decay to 1 and 10% of online measurements, respectively (Fig. 1B). As most studies quantifying PM-mediated ROS formation and OP use offline filter analyses (Fig. 1A), where particle samples are analyzed days or even months after sample collection, it can be concluded that most literature values severely underestimate SOA contributions to OP_T_ and particle-bound ROS_T_.

**Fig. 2.**
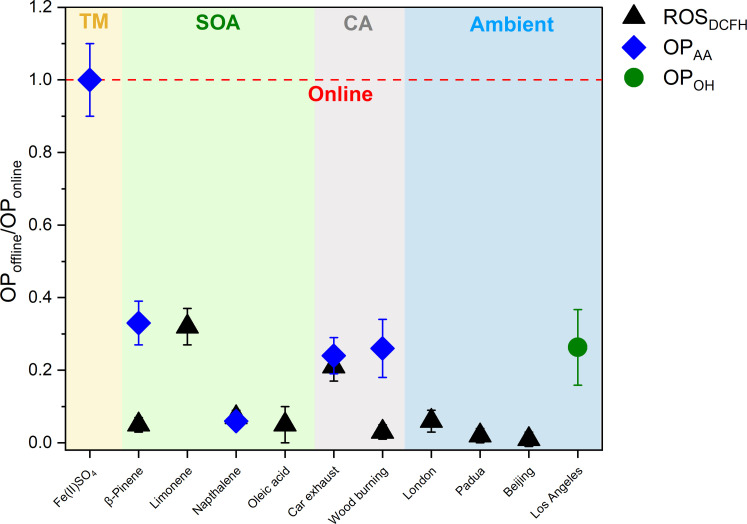
A substantial fraction of ROS and OP activity in aerosol particles decays before offline analysis. Ratio of filter-based offline to online quantification, relative to online measurements (red dashed line) of ROS_DCFH_ (black triangle), OP_AA_ (blue diamond), and OP_OH_ (green circle) for a range of particle types: transition metal particles [Fe(II)SO_4_, TM, light orange], SOA (light green), aged combustion-generated aerosol particles (CA, light gray), and ambient PM_2.5_ (OP_AA_ and ROS_DCFH_) and PM_1_ (OP_OH_) from locations in Europe, Asia, and the US (light blue). We consistently observed 60 to 99% loss of ROS and OP using filter-based offline methods depending on particle type, location, and assay. It should be noted that online ROS_DCFH_ and OP_AA_ were quantified using the OPROSI (*30*) and the OOPAAI (*25*, *29*), and OP_OH_ was quantified using a spot sampler, an approach that collects particles directly into an aqueous solution containing the terepthalate probe (TA) to quantify OH radical formation. More details on this alternative direct-to-reagent sampling approach are presented by Taghvaee *et al.* (*49*). Time delays between filter sampling and offline analysis are presented in table S1. Data for limonene SOA and oleic acid SOA are from Gallimore *et al.* (*79*) and Fuller *et al.* (*23*), respectively. Data from β-pinene SOA and naphthalene SOA are from Campbell *et al.* (*45*).

Beyond observing the substantial loss of short-lived components contributing to OP_T_ and particle-bound ROS_T_ activity in SOA particles, we also demonstrate that offline analysis underestimates OP_T_ and ROS_T_ in a broad range of particles with differing composition. We quantified ROS_DCFH_, OP_AA_, and hydroxyl radical formation (OP_OH_) with online instruments and filter-based offline methods, sampling anthropogenic and biogenic SOA, metal particles, aged car exhaust, and wood combustion emissions, as well as ambient PM_2.5_ from locations in Europe, Asia, and the US (Fig. 2 and table S1). Using offline filter-based methods, we observed 60 to 99% loss of ROS_DCFH_, OP_AA_, and OP_OH_, depending on particle type, ambient sampling location, and assay used. Not only do we observe the short-lived nature of OP and ROS in laboratory experiments but also in particles characterized at urban locations around the world (London, Padua, Beijing, and Los Angeles; Fig. 2). The exception was Fe(II)SO_4_ particles, where comparable values were obtained for online and offline OP_AA_ measurements over an experimental timescale of approximately hours. This is expected because Fe(II)SO_4_ particles are chemically stable under the experimental conditions used here (fig. S4). The short-lived OP and ROS fractions of all other particles sampled vary widely, making it challenging to accurately estimate ROS_T_ and OP_T_ from offline measurements.

**Fig. 3.**
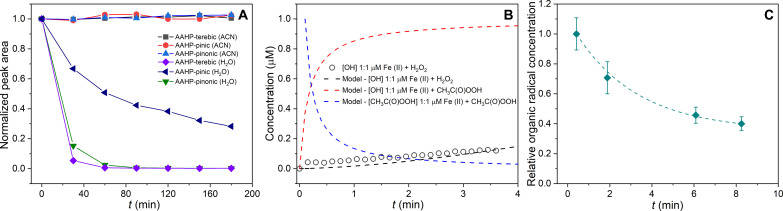
Fast decay of OP- and ROS-active components in aerosol particles. Compound-specific analyses demonstrate that labile and reactive components are likely important contributors to the short lifetime of OP_T_ and ROS_T_ on the order of minutes or hours. (**A**) Organic peroxides contributing to OP_T_ and ROS_T_ have widely variable lifetimes from minutes to several hours. AAHPs have substantially shorter lifetimes in water (90% water and 10% ACN) than in organic medium (ACN, 100%) (*75*). (**B**) Modelled OH radical formation (red dashed line) from the aqueous reaction of 1 μM Fe(II) and 1 μM peracetic acid [CH_3_C(O)OOH], which occurs much faster than the classic Fenton reaction between 1 μM Fe(II) and 1 μM H_2_O_2_ (black circles). Kinetic modeling predicts rapid CH_3_C(O)OOH decay within minutes in an aqueous medium in the presence of Fe(II) (blue dashed line). (**C**) Organic radicals (green diamonds) also decrease in concentration in α-pinene SOA particles within minutes (*22*).

To understand this significant, fast, and variable decay of OP_T_ and ROS_T_, a molecular-level perspective of aerosol processes in the atmosphere as well as during sample collection and analysis is necessary. The concentrations of metals in aerosol particles, contributing to OP_T_ by generating peroxides and radicals or directly depleting antioxidants (*32*), are likely relatively stable for several hours or days. However, organic radicals and peroxides have substantially shorter but highly variable lifetimes from a few minutes to hours, as shown in Fig. 3 (*21*, *22*, *31*, *33*–*36*). The chemical structures of these unstable compounds as well as the chemical environment are important factors that determine their lifetimes in aerosol particles. Figure 3A illustrates that hydroperoxides hydrolyze in aqueous environments (e.g., during particle collection and extraction in aqueous media as used in most offline studies) with lifetimes that range between a few minutes to an hour depending on their structure. However, in an organic medium (e.g., in organic aerosols), they can remain stable over several hours or longer (Fig. 3A). Other aerosol components also affect the lifetime of peroxides—the presence of redox-active transition metals like iron can accelerate the decay of peroxides (*37*) (e.g., peracetic acid, CH_3_C(O)OOH, an organic peracid) to generate OH radicals by more than three orders of magnitude compared to Fenton-chemistry involving H_2_O_2_ (Fig. 3B). OH radicals, which are the most reactive ROS components themselves, are highly reactive and are likely to evade quantification in offline analysis, particularly when particles are extracted in aqueous solution before introduction to the reagent (*31*). Fresh SOA can contain very high concentrations of peroxides, up to 30 to 80% (*9*), and therefore their fast but variable decomposition could explain the large and variable fraction of short-lived ROS and OP observed in Fig. 2. Total radical concentrations in SOA decay with similar timescales of a few minutes, while a smaller fraction is longer lived (Fig. 3C). The results presented in Fig. 3 illustrate that two important classes of chemical species (peroxides and radicals) that contribute to particle-bound ROS and OP have lifetimes as short as a few minutes, especially in aqueous media.

### Biological effects of OP_T_ and ROS_T_ of particles

The variable but consistent underestimation of the OP_T_ and ROS_T_ burden of PM using traditional filter–based methods adds uncertainty to comparisons made between OP and toxicological end points, e.g., in studies where particles are deposited on cell cultures. To account for the fast decay of OP_T_ and ROS_T_, it is important that particles are deposited directly from a continuous air flow onto cells residing at the ALI to avoid underestimating potential toxicity effects of short-lived ROS and OP (*38*, *39*). In addition, the effects of gaseous components need to be removed to assess particle effects only (see the Supplementary Materials), although synergistic toxicity effects of particles and gases could also occur (*10*).

**Fig. 4.**
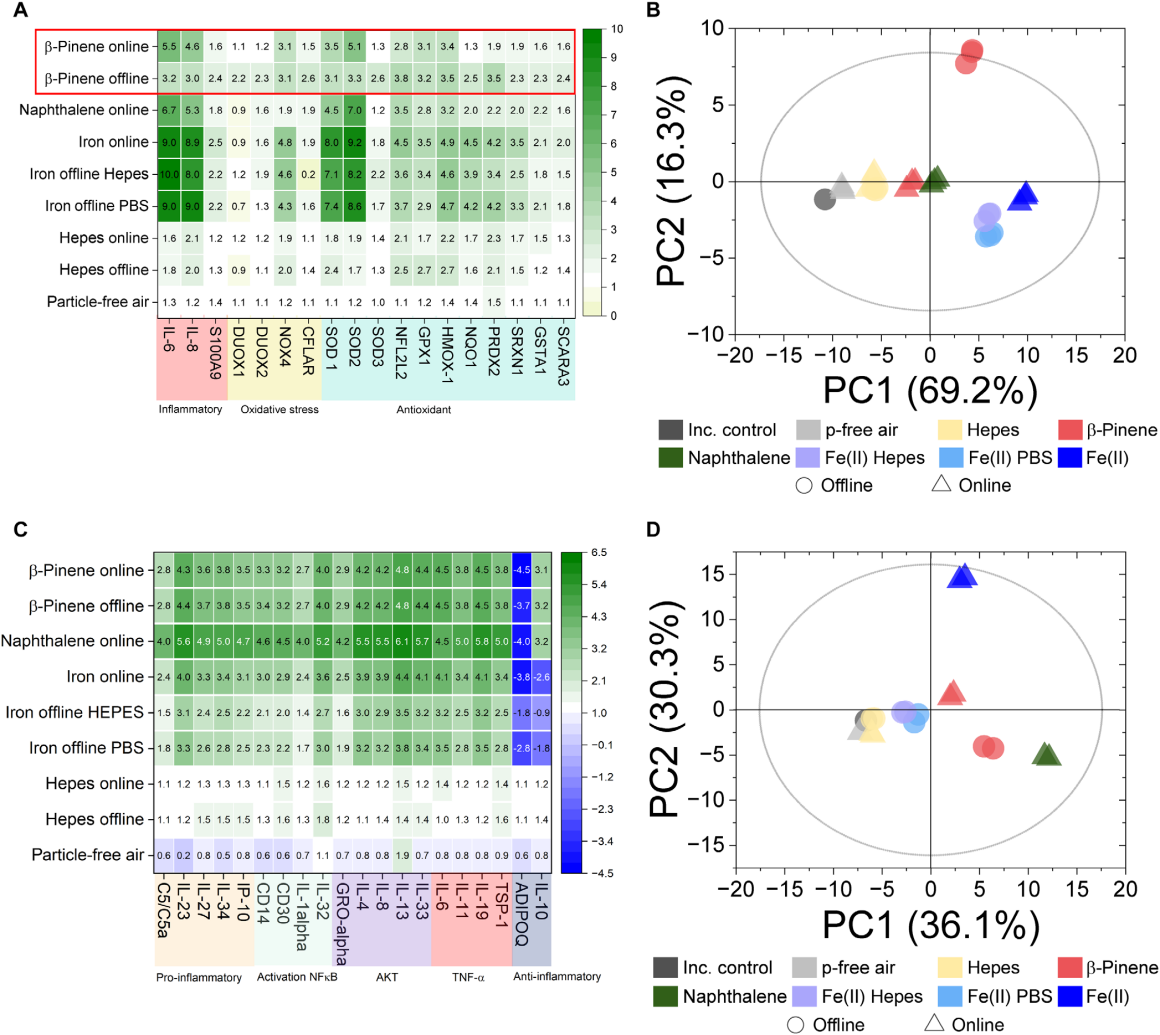
Biological responses of reconstituted HBE after online and offline particle deposition. Responses of reconstituted HBE after online and offline particle deposition. (**A**) Gene activation [shown as fold change compared to incubator control (inc. control)] after deposition of biogenic SOA (β-pinene), anthropogenic SOA (naphthalene), iron(II) sulfate [Fe(II)SO_4_], and Hepes-buffer particles, illustrated with a heatmap and (**B**) PCA. (**C**) Protein expression of all particle types mentioned above and (**D**) PCA. Controls: p-free air and inc. control. It should be noted that heatmaps in (A) and (C) focus on the 18 genes and 20 proteins that showed the highest fold change, respectively, whereas the PCA score plots in (B) and (D) consider all measured 20 genes and 105 proteins. The *Q* values are as follows: for genes (B), Q2 (1) = 0.677 and Q2 (2) = 0.492; for proteins (D), Q2 (1) = 0.221 and Q2 (2) = 0.405. Only for β-pinene SOA were strong differences observed for online/offline gene activation, consistent with the fast decay of OP_T_ in these particles. In Fe(II)SO_4_ particles, OP_T_ is high but stable and, thus, no significant gene activation differences for online/offline exposure are observed. Effects of Hepes particles (serving as negative controls with no OP and ROS activity) and comparison between iron-PBS and iron-Hepes are discussed in the Supplementary Materials. Controls: p-free air and inc. control.

We show here that direct online organic particle deposition at the ALI induces significantly different biological effects compared to offline deposition, which can be attributed to the fast decay of OP_T_ in organic particles, indicating that OP_T_ could be a key parameter explaining particle toxicity. Figure 4 compares gene activation in ALI cultures of reconstituted human bronchial epithelia (HBE) (*40*–*42*) after online and offline (i.e., exposing cells to aqueous particle extracts collected on filters) particle deposition. The investigated particles [biogenic and anthropogenic SOA, Fe(II)SO_4_, and Hepes-buffer particles, where the latter is considered to be nontoxic to cells (*43*)] differ in their OP_T_ and ROS_T_ stability and concentration and, therefore, in their expected toxicity. Figure 4A illustrates that online deposition of biogenic SOA particles induces a much stronger gene activation of the immune system [interleukin-6 (IL-6) and interleukin-8 (IL-8)] and some oxidative stress markers [superoxide dismutase 2 (SOD2)] compared to offline exposure, expressed as fold changes compared to incubator controls (Fig. 4A, red rectangle). In contrast, for offline exposure of cells to SOA, a wide range of antioxidative and oxidative-stress genes is slightly more activated than following online deposition. These strong online/offline differences in biological responses were also illustrated using principal component analysis (PCA) (Fig. 4B) and may be explained by the lower OP_T_ activity during offline β-pinene SOA particle deposition (compared to online deposition), triggering only a moderate expression of antioxidant genes without initiating a strong inflammatory response. In contrast, after the deposition of substantially more OP-active particles during online exposure, the antioxidant defenses appear insufficient, unable to scavenge ROS deposited by the particles, and thus, inflammatory genes (i.e., IL-6 and IL-8) are activated as a second defense line of the cell response. Previous studies have shown that air pollutants can enhance intracellular ROS levels by suppressing antioxidants (*38*, *39*, *44*). PCA of protein expression (Fig. 4D) supports this finding for β-pinene SOA, showing differences of protein expression that are indicative of oxidative stress and inflammatory pathways between online and offline cell exposure. These results clearly demonstrate that online and offline exposures of SOA cause substantially different cell responses. To identify particle toxicity pathways because of particle exposure, realistic online cell exposure methodologies are required for evaluating the adverse effects of chemically unstable particle components, especially in the organic fraction, an often-dominant component of ambient PM. It has been demonstrated that while oxidative stress responses lead to activation of the antioxidant machinery, such as SOD2-, Nrf2 (nuclear factor erythroid 2–related factor 2)–, and ARE (antioxidant response element)–mediated transcriptional responses, PM exposure reduced these mechanisms, especially at higher doses (*38*, *39*, *44*). Short-lived SOA components like peroxides and radicals (Fig. 3) could be key drivers of this difference; however, other as-yet unidentified labile components could also contribute to the differences observed between online and offline exposures and merit further investigation in future studies.

In contrast to SOA, for Fe(II)SO_4_ particles, OP_T_ was stable over the experimental time frame used in this study (Fig. 2). Fe(II) is known to generate high concentrations of ROS in aqueous media through Fenton-like chemistry, while Fe(II)SO_4_ is chemically stable during offline handling over timescales in these experiments (fig. S4). Thus, no significant differences in cellular responses are observed for gene activation when comparing online/offline Fe(II)SO_4_ particle deposition (Fig. 4), in agreement with OP_T_ measurements (Fig. 2). However, we found that the adverse effects of Fe(II) on cells can be severely underestimated and suppressed in offline particle exposures, especially as observed for cytotoxicity and protein expression (Fig. 4 and fig. S5). This is likely due to interactions (e.g., complexation and inactivation) of iron with buffer ions in solution, emphasizing again that online cell exposure experiment designs, where cells are kept under ALI conditions without buffer, are essential to decipher toxicity pathways of atmospheric particles.

Comparing only the online deposition experiment results, we show that iron(II) particles and anthropogenic naphthalene SOA induce enhanced inflammatory and oxidative stress pathways compared to biogenic β-pinene SOA on gene activation and protein expression (Fig. 4, A and C) and cytotoxicity level (fig. S5). This is consistent with OP_T_ measurements, where iron(II) prompts the highest response followed by anthropogenic and biogenic SOA (*25*, *45*).Higher toxicity of anthropogenic SOA was observed in earlier studies and might be attributed to OP-active components of oxidized aromatic compounds like quinones (*46*–*48*). Despite comparable doses of naphthalene SOA compared to other conditions (table S2), we observe elevated lactate dehydrogenase (LDH) release compared to other exposure conditions (fig. S5), and therefore, gene and protein expression data should be interpreted with caution.

## DISCUSSION

Previous attempts to quantify OP and ROS with online and semionline instruments have been hindered because of instrument design limitations, where particles are collected in water for several minutes, in some cases, up to an hour before quantification (*28*, *49*–*52*). We clearly demonstrate (Figs. 1 to 3) that such time delays between particle collection and OP or ROS quantification cause a significant underestimation of OP_T_ and ROS_T_. A total of 60 to 99% of OP_T_ and ROS_T_ has a short lifetime of a few minutes to a few hours in many particle types (i.e., SOA, car exhaust emissions, wood burning emissions, and ambient PM_2.5_). These large online/offline differences were observed with three acellular methods (ROS_DCFH_, OP_AA_, and OP_OH_), demonstrating that these results are robust and not caused by methodological artifacts associated with a specific assay. OP_DTT_ activity also decays by more than 60% within 2 hours in laboratory-based offline experiments using β-pinene SOA, as illustrated in Fig. 1A. At present, no online instrument that uses a direct-to-reagent sampling approach is available for comparison, so while we observe the same decay in OP_DTT_ as for OP_AA_ and ROS_DCFH_ in the laboratory, future work should investigate this effect in ambient studies. In traditional filter–based analyses, this short-lived OP and ROS fraction is not quantified because of the delay, typically of a day or longer, between particle sampling and OP and ROS quantification. We show that short-lived and reactive peroxides and radicals are likely responsible for this fast decay of OP_T_ and ROS_T_ in particles. In the atmosphere, radicals and peroxides are formed directly or indirectly via oxidation and photochemical processes (*21*, *53*, *54*). Thus, they are continuously generated in the gas phase and condense onto particles or are generated directly in particles during their entire atmospheric lifetime, possibly maintaining steady-state concentrations of OP_T_ and ROS_T_ in airborne particles (*21*, *54*). It was recently shown that OH radicals and H_2_O_2_ (*55*) are spontaneously generated on the surface of aqueous droplets, which may be another pathway for continuous ROS generation in aerosol particles (*56*). The large fraction of short-lived ROS and OP, especially in ambient samples shown in Fig. 2, suggests that in the atmosphere, continuous OP and ROS atmospheric formation processes may occur. Continuous formation of short-lived ROS and their precursors via oxidation and photochemistry or other atmospheric aging processes, which stop once particles are collected on a filter, would explain the large differences between online and offline OP_T_ and ROS_T_ in ambient samples, as most PM is formed many hours to days before analysis with our online instruments in the ambient. In addition, this constant generation of ROS and OP may explain the increased inflammation responses in reconstituted human bronchial epithelia during online particle deposition experiments (Fig. 4).

Besides the capability to accurately quantify short-lived ROS and OP, another advantage of online instruments is that they provide highly time-resolved data, capturing transient emissions and atmospheric processes (e.g., diurnal profiles, changes in wind direction, and transient sources contributing to OP_T_ and ROS_T_) that occur on timescales of minutes (*25*, *57*). Understanding the influence of these processes on OP_T_ concentrations is essential to quantify source contributions and the atmospheric conditions that lead to increased OP_T_ and establish the relationships with adverse health outcomes, which can lead to more targeted air pollution abatement policies. Offline assays lack this time-resolved capacity as samples are typically collected for 24 hours and are not immediately analyzed for their ROS or OP activity.

Only a few studies have attempted to compare biological effects of online versus offline particle exposure. However, these studies typically involved either metal nanoparticles or fresh soot particles, where no or only very limited chemical changes of particle composition are expected if deposited online or offline on cell cultures (*58*–*64*). The few studies that investigated the effects of organic or ambient particles in online/offline experiments all focused on the combined toxicity of the gas and particle phase and observed larger effects of gas-particle mixtures (online exposure) compared to particles only (offline exposure) (*61*, *65*, *66*). However, these studies did not attempt to identify online/offline toxicity differences of particles only. They attributed the increased biological effects in online versus offline experiments to gaseous components, filter sampling artifacts, and difficulties of dose estimation during offline experiments. These aspects may also influence the results presented in Fig. 4, but the large differences in OP_T_ between online and offline exposures as a driver of particle toxicity have not previously been considered before in particle toxicity evaluations.

It is becoming increasingly clear that particle composition plays a significant role in particle-mediated health effects. OP measurements are emerging as a key metric that provides a crucial link between particle composition and toxicity. Thus, robust quantification of this metric, including the contribution from highly reactive components, is essential to confidently identify links of OP_T_ and ROS_T_ with PM sources and health outcomes. The fast decay and, especially, the high variability of the short-lived OP fraction in a wide range of particles implies that online OP instruments, which use a direct-to-reagent sampling approach, should be included in future air monitoring networks, possibly in parallel with offline OP analyses. This will be crucial for identifying links of OP_T_ with particle composition, toxicity, and health effects.

## MATERIALS AND METHODS

### Experimental design

#### Reagents

The following reagents were purchased from Merck: l-ascorbic acid (≥99%), l-dehydroascorbic acid, H_2_O_2_ solution (3%), Chelex 100 sodium form, 1 M HCl solution, 0.1 M NaOH solution, 1 M phosphate-buffered saline (PBS) solution, Hepes (≥99.5%), Fe(II)SO_4_ (≥99%), *o*-phenylenediamine (OPDA; ≥99.5%), β-pinene (98%), naphthalene (98%), horseradish peroxidase (HRP; type VI), 2,7-dichlorofluoroscein diacetate (98%), trichloroacetic acid (TCA; ≥99.0%), 2-amino-2-(hydroxymethyl)-1,3-propanediol (tris base, 99.9%), EDTA (≥99.0%), 5,5′-dithio-bis-(2-nitrobenzoic acid) (DTNB, 99%), and DTT (97%).

Disodium terepthalate and 2-hydroxyterepthalic acid for OP_OH_ measurements were purchased from TCI (US). All aqueous solutions were prepared using water obtained from a Merck Synergy high-purity water unit (resistivity ≥18.2 megohms cm^−1^). High-purity water was further purified by flowing through a 10-cm column packed with Chelex resin at a flow rate of 1 drop/min to minimize background contributions from transition metals (*25*).

#### SOA generation using the organic coating unit (OCU)

A flow through reactor [organic coating unit (OCU)] (*67*) was used to generate SOA. In short, the OCU maintains a steady concentration of the gas phase volatile organic compound (VOC) precursor, which is maintained via a proportional-integral-derivative voltage feedback. For β-pinene SOA production, 1 ml of β-pinene was placed in a reservoir at room temperature with a steady gas flow passed over the surface. For naphthalene SOA production, 1 g of naphthalene was placed in the reservoir and heated to 80°C using a water bath to volatilize the naphthalene into the gas phase. The VOCs were then passed through a cylindrical quartz photooxidation chamber (76-ml volume) surrounded by five low-pressure mercury lamps (4-W ultraviolet (UV) C with 254- and 185-nm emission lines, type GPH212T5VH/2, Heraeus, Germany), which produce both O_3_ and OH radicals via the photolysis of O_2_ under humid conditions. Only one UV lamp was turned on for all online photooxidation experiments presented in this study, resulting in a maximum O_3_ concentration of 2 × 10^13^ molecules cm^−3^ and an estimated OH concentration in the chamber of ~1 × 10^9^ molecules cm^−3^ in the absence of VOCs, with a constant gas flow of 1 liter/min through the oxidation chamber. OH concentrations were estimated on the basis of the rate of consumption of gas phase naphthalene in the photooxidation chamber. We expect predominantly OH-initiated β-pinene SOA because of the greater rate constant associated with OH reaction (7.9 × 10^−11^ molecules cm^−3^ s^−1^) at the exocyclic double bond compared to O_3_ (1.5 × 10^−17^ molecules cm^−3^ s^−1^), whereas naphthalene oxidation proceeds only through OH-initiated oxidation (*68*). All experiments were performed at a 70 ± 20% relative humidity in the chamber, which was maintained using a humidifier in the OCU. Particles generated using the OCU were then analyzed using a range of instrumentation as described in fig. S1.

#### Generation of Hepes and Fe(II) particles

Fe(II)SO_4_ and Hepes particles were generated with a homebuilt nebulizer using aqueous solutions of 4 mM Fe(II)SO_4_ and Hepes buffer, respectively. The aerosol was dried by passing through a silica gel denuder after the nebulizer. The aerosol was then either analyzed directly with the online oxidative potential ascorbic acid instrument (OOPAAI) or online particle-bound ROS instrument (OPROSI) for online analysis or collected on a filter (Teflon) and analyzed offline. Offline analysis using either the AA or DCFH assay was performed using the same chemical conditions as the OOPAAI and OPROSI (see fig. S1).

#### Generation of car and wood burning particles

Aerosol particles were collected from the exhaust of either a EURO6 gasoline car (Škoda Scala 2021) or a residential wood stove (Aduro 9.3). The passenger car was operated in a Rototest VPA-RX3 2WD chassis dynamometer under steady-state driving conditions and by using a 95 E10 gasoline as fuel. Particle emissions were characterized for a 60-min driving cycle, which included idling and 50, 100, and 80 km/hour velocities, each 15 min. The residential stove was fired with beech logs, and every 35 min, a new wood batch was added to the stove for a total duration of 3.5 hours (i.e., six batches of beech logs) (*69*). The exhaust aerosol was passed through a heated sampling line where particle concentrations and temperature were reduced by a two-step dilution system with a porous tube and ejector diluter (1:17 for the car exhaust and 1:60 for the wood stove combustion). To simulate atmospheric aging, the diluted, freshly emitted aerosol was passed through the photochemical emission aging flow tube reactor (*70*) to simulate atmospheric aging by ozone and OH radicals. For the online OP_T_ and ROS_T_ measurements, the OOPAAI and OPROSI were connected after the photochemical emission aging flow tube reactor and, for the wood stove experiments, an additional porous tube diluter was connected and set to a dilution ratio of 1:3. For the offline analysis, the same aerosol was collected on a quartz filter (PALLFLEX, Tissuquartz 2500-QAT-UP) and analyzed 24 hours after storage at 4°C with the same chemical assay as used in the OOPAAI and OPROSI, respectively.

#### Online particle-bound ROS instrument (OPROSI; ROS_DCFH_)

The functionality, design, and operating procedure for the OPROSI have been extensively described by Wragg *et al.* (*30*) and Fuller *et al.* (*23*). Briefly, the aerosol is continuously drawn into the instrument via an aerosol conditioning unit, which consists first of a stainless-steel cyclone (2.5-μm cutoff at 5 liter/min, URG-2000-30E-5-2.5-S, URG) and charcoal denuder, before entering a homebuilt particle sampler. Particles are collected onto a filter sprayed with an aqueous solution of HRP in 10% PBS buffer at 1 ml min^−1^, which immediately extracts and reacts with ROS present in the particles and is collected in a 1-ml liquid reservoir. The HRP/particle extract solution is then immediately mixed with DCFH, which is subsequently oxidized to form a fluorescent product dichlorofluorescein by HRP in a reaction bath maintained at 37°C for 10 min. Dichlorofluorescein is then quantified via fluorescence spectroscopy. The fluorescence response of the instrument is calibrated with known concentrations of hydrogen peroxide (H_2_O_2_), and thus, ROS concentrations are expressed in H_2_O_2_ equivalent concentrations per unit air volume (m^−3^) or per unit particle mass (μg^−1^). The assay has demonstrated sensitivity in particular to hydrogen peroxide and organic peroxides (*23*, *30*). The direct-to-reagent sampling and high time resolution of this instrument therefore are particularly sensitive to short-lived ROS components, which react within seconds after sampling (*23*, *30*).

#### Online oxidative potential ascorbic acid instrument (OOPAAI, OP_AA_)

The OOPAAI is described in detail by Utinger *et al.* (*25*), and a brief description is given here. The OOPAAI measures OP_AA_ by quantifying the formation of dehydroascorbic acid (DHA), an oxidation product of AA. DHA is reacted with OPDA, forming a fluorescent product 3-(1,2-dihydroxyethyl)-fluoro-[3,4-*b*]quinoxaline-1-one (DFQ), which is quantified using fluorescence spectroscopy.

Aerosol particles are continuously collected for online OP_AA_ analysis using a commercially available particle-into-liquid sampler (Brechtel, US), where the wash flow was modified to contain the AA reagent, ensuring direct-to-reagent particle sampling, rapid extraction of aerosol particle components, and reaction with AA (200 μM AA buffered to pH 6.8 with 20 mM Hepes). The sample is washed off the impactor with a flow rate of 60 μl min^−1^, and the resulting AA/particle extract solution is reacted for 20 min at 37°C. The solution is then mixed with OPDA in 0.1 M HCl at a flow of 90 μl min^−1^ and pumped into another reaction coil at room temperature for 2 min, where the DHA + OPDA reaction occurs, forming the fluorescent product DFQ. The concentration of DFQ is then monitored using a homebuilt flow through fluorescence cell, which consisted of a modified flow-through quartz cuvette (Hellma Analytics). DFQ is excited by a high-power UV light-emitting diode (LED; Roithner Lasertechnik, type UVLED-365-330-SMD) at 365 nm via an optical fiber (Thorlabs; 1500 μm; numerical aperture, 0.39). The fluorescence emission light is then collected through a collimating lens (Ocean Insight) via an optical fiber (Thorlabs; 1500 μm; numerical aperture, 0.50) and detected using a spectrometer (Ocean Insight, QePro). The OOPAAI is calibrated using known concentrations of DHA, and hence, the OP is expressed in terms of nanomole DHA per unit volume (m^−3^) or unit mass (μg^−1^).

#### Time-resolved measurements of ROS and OP decay of SOA on filters

To determine the decay kinetics of ROS and OP, offline experiments were conducted with biogenic SOA (β-pinene). Particles were collected on quartz filters for 100 s with a high β-pinene SOA mass concentration (80 mg m^−3^) with atmospherically representative composition (*67*) produced by the OCU (see above) (*67*). After the OCU, an additional flow tube with an ozone flow was added to oxidize the VOC that was not oxidized in the OCU. The high aerosol mass concentrations were necessary to minimize the collection time (i.e., 100 s) and therefore capture short-lived OP-active aerosol components for offline ROS and OP analyses. The filters (47-mm quartz, Whatman) were then extracted after storing for different time intervals at room temperature [immediately after sampling (2 min), then at 4 and 22 min, and 2, 24, 48 and 168 hours; see Fig. 1A] and immediately analyzed after extraction with an offline AA, DCFH, and DTT assay. AA and DCFH chemical procedures (e.g., concentrations, pH, etc.) were identical to those used in the respective online instrument (i.e., OOPAAI or OPROSI) (see above), and the DTT protocol follows that of Cho *et al.* (*71*), as described in more detail below.

For the offline AA measurements, the filters were extracted directly in 3 ml of 200 μM AA buffered with 20 mM Hepes and then vortexed for 3 min. The slurry was then filtered with a syringe filter (polytetrafluoroethylene; pore size, 0.45 μm; Agilent), and 900 μl of the extract was reacted for 10 min for the decay experiments (Fig. 1) and 20 min for all other offline experiments (Fig. 2) in a 37°C heating bath. Subsequently, 100 μl of OPDA dissolved in 0.1 M HCl was added and reacted for an additional 3 min at room temperature before immediate analysis with fluorescence spectroscopy. The excitation wavelength was 365 nm using an LED, and the fluorescence emission measured was 430 nm.

For the offline DCFH measurements, the filter was extracted in 3 ml of water and vortexed for 3 min before passing the aqueous sample through a syringe filter. The sample (0.833 ml), 1 ml of 10 μM HRP in 10% PBS, and 1 ml of 10 μM DCFH in 10% PBS were mixed and incubated at 37°C for 20 min before analysis. The excitation wavelength was 470 nm using an LED, and the emission was measured at 520 nm.

The DTT protocol follows that of Cho *et al.* (*71*). TCA was prepared as a 10% solution (20 g of TCA in 200 ml of water). EDTA and tris(hydroxymethyl)amino-methane were mixed in the following ratio: 20.2 g of tris(hydroxymethyl)amino-methane and 2.45 g of EDTA in 23.7 2 ml of aqueous HCl (1 M; 226.28 ml of chelexed ultrapure water). The phosphate buffer (0.2 M) at pH 7.4 was prepared with 77.8 ml of NaH_2_PO_4_ (1 M) and 22.2 ml of K_2_HPO_4_ (1 M). DTNB (Ellman’s reagent) was prepared using 59.46 mg of DTNB in 7.5 ml of 0.2 M PBS and 7.5 ml of Milli-Q water, which has undergone Chelex treatment. DTT is prepared using 30.8 mg of DTT in 20 ml of 0.2 M PBS for a 10 mM DTT stock solution. For the working solution, 1.125 ml of the stock solution was diluted with 48.875 ml of PBS. Filter extraction is performed in the same way as described for AA above. Then, 2.5 ml of the extract is mixed with 2 ml of the working solution of DTT. The vial reacts for 30 s in the dry heater (38°C), and 0.6 ml is transferred to 0.6 ml of TCA. This vial must already be prepared before proceeding to minimize transfer time. This step is repeated after 2, 5, 12, 22, and 32 min to get the slope of the decay. For absorbance measurements, a quartz cuvette is filled with 800 μl of the final DTT solution and absorption is measured at 412 nm with an integration time of 70 ms. The variability for triplicates was less than 5%.

The fluorescence signal of the AA and DCFH assays were measured with a spectrometer (QePro, Ocean Insight). For the offline DTT measurements, a deuterium lamp (DH-mini, Ocean Insight) was coupled via an optical fiber (600-μm diameter, Ocean Insight) to a cuvette holder (4 Way CUV Cuvette Holder, Ocean Insight) and from there directly to a spectrometer (QePro, Ocean Insight). The fibers were connected with a collimating lens (SMA, Ocean Insight) to the cuvette holder.

#### Ambient online and offline quantification of ROS_DCFH_ and OP_AA_

Online ROS_DCFH_ and OP_AA_ concentrations in ambient aerosol particles in London, Padua, and Beijing were determined using the OPROSI and OOPAAI described by Wragg *et al.* (*30*) and Utinger *et al.* (*25*), respectively.

In all three cities, samples were collected at a city center location. The sampling site in London was at the Marylebone Road urban traffic monitoring station (50°31′21″N, 0°09′17″W) from 6 August to 28 August 2019, in Padua at the top floor of the Department of Chemical Sciences of the University of Padua (45.4067°N, 11.8772°E) from 8 January to 29 January 2020, and in Beijing at the Institute of Atmospheric Physics (39°58′28″N, 116°22′15″E) for 7 days between 26 May and 2 June 2017.

For ROS_DCFH_ and OP_AA_ offline analysis at these three locations, high volume sampler PM_2.5_ samples were collected on quartz microfiber filters for 24 hours and stored at <−22°C until analysis. Filter punches were extracted in 5 ml of water and filtered using 0.45- and 0.2-μm pore size filters. More details are given by Steimer *et al.* (*72*), Campbell *et al.* (*16*), and Tong *et al.* (*73*).

#### Online (direct-to-reagent) and offline quantification of OH radicals (OP_OH_) in ambient PM

Hydroxyl radical production (OP_OH_) from PM_2.5_ was quantified using a direct-to-reagent sampling approach and filter-based (offline) sampling approach. OP_OH_ was determined using the terephthalate (TA) probe (*74*). TA reacts selectively with OH to produce the highly fluorescent product 2-hydroxyterepthalate (hTA), which is then detected at *l*_em_ = 420 nm (*l*_ex_ = 320). A 325-nm peak emission LED (M325F4, Thorlabs) was coupled to a cuvette cell (CVH100) using quartz cuvettes to ensure efficient UV transmission and a QEpro (Ocean Insight) spectrometer to facilitate fluorescence detection. Direct-to-reagent samples collected at the UCLA (University of California, Los Angeles) campus are obtained using a spot sampler method, as described in detail by Taghvaee *et al.* (*49*), and were compared to PM_2.5_ filters collected in parallel for typically ~7 hours at a rooftop sampling site at UCLA Mathematical Sciences Building (34°04′10.1″N, 118°26′35.3″W, 32 m above the ground level, between 21 February and 5 March 2023), located near western Los Angeles edge, ~8 km from the Pacific Coast. Filtered samples were frozen at −20°C for 2 weeks and then analyzed using the same chemical procedure as the direct-to-reagent approach to determine the decay of OP_OH_ on filters.

#### *Quantification of OH production from Fe(II)*-H_2_O_2_

OH radicals generated from the reaction of hydrogen peroxide (H_2_O_2_)and Fe(II) were quantified using the TA probe (*74*). Reactions were performed by mixing H_2_O_2_ prepared in Milli-Q water adjusted to pH 4 using 0.1 M H_2_SO_4_ with Fe(II)SO_4_, also at pH 4. Reactions were performed at equimolar concentrations of 1 μM H_2_O_2_ and 1 μM Fe(II)SO_4_. Excess aqueous TA (10 mM) reacts with OH to produce the highly fluorescent product hTA, which was then detected at *l*_ex_/*l*_em_ = 320/420 nm. Measurements of hTA were done using a fluorometer (Lumina, Thermo Fisher Scientific).

#### Synthesis and hydrolysis properties of atmospherically relevant organic peroxides

It is well known that the ozonolysis of alkenes produces stabilized Criegee intermediates (SCIs), which can further react with water, alcohols, aldehydes, and carboxylic acids to form organic peroxides (*21*). Among those different SCI-involved bimolecular reaction channels, α-acyloxyalkyl hydroperoxides (AAHPs), formed through SCI reactions with carboxylic acids, are considered a major class of organic peroxides in the atmosphere (*75*).

Because of the limited availability of organic peroxide standards, we synthesized AAHP standards through liquid phase ozonolysis (*76*). A 10-ml acetonitrile (ACN) solution containing 1 mM α-pinene and carboxylic acid mixtures (including terebic acid, *cis*-pinic acid, and *cis*-pinonic acid, each 20 μM) was prepared in an impinger. Then, O_3_ (~500 parts per million with a flow rate of 100 ml min^−1^) was bubbled through this solution for 15 min, which leads to the formation of SCIs via the reaction of O_3_ and α-pinene. SCIs are immediately scavenged by added carboxylic acids to form corresponding AAHPs. A control experiment was performed for the same α-pinene bulk solution in the absence of carboxylic acids, allowing the unambiguous identification of AAHPs in the extracted ion chromatogram through LC–high-resolution mass spectrometry (LC-HRMS) analysis. The three selected carboxylic acids are known oxidation products of α-pinene. Thus, the synthesized AAHPs, here designated as apSCI-terebic (C_17_H_26_O_7_), apSCI-pinic (C_19_H_30_O_7_), and apSCI-pinonic (C_20_H_32_O_6_), are likely present in atmospheric monoterpene-derived SOA.

The synthesized AAHP solutions were separated and analyzed by LC-HRMS, which consists of an ultraperformance LC unit (ACQUITY UPLC I-Class, Waters) coupled with a high-resolution mass spectrometer (Orbitrap Q Exactive Plus, Thermo Fisher Scientific). Analytes were separated using a Waters HSS T3 UPLC column (100 mm by 2.1 mm, 1.8 μm) at a temperature of 40°C. The mobile phases include the following: (A) 0.1% formic acid in water and (B) methanol. The gradient elution was performed by the A/B mixture at a total flow rate of 0.3 ml min^−1^ for 30 min: 0 to 1 min at 99.9% A, 1 to 26 min with a linear gradient to 99.9% B, 26 to 28 min held at 99.9% B, and 28 to 30 min back to the initial condition at 99.9% A for column re-equilibration. The synthesized AAHPs were detected as sodium adducts [M + Na]^+^ in positive mode. To compare the stability of AAHPs in water versus the nonpolar organic solvent ACN, water was added to the AAHP solutions (*v*/*v* = 1/9 ACN/water) in an amber vial, followed by the same LC-HRMS analysis. The autosampler temperature during all LC analyses was set at 20°C.

#### Kinetic modeling of OH formation and CH_3_C(O)OOH reaction with Fe(II)

The kinetic model describing aqueous Fe(II) and CH_3_C(O)OOH chemistry is presented by Campbell *et al.* (*37*) and Shen *et al.* (*32*). It includes 85 individual reactions describing the reactions between Fe(II) and peracetic acid (dark chemistry), as well as inorganic aqueous Fe(II)/Fe(III)/Fe(IV) chemistry. It also includes aqueous ROS (ROS-OH, HO_2_, H_2_O_2_, and O_2_^·−^) reactions, TA chemistry for measuring OH, and photolysis reactions of Fe(OH)_2_^+^, H_2_O_2_, and peracetic acid. The kinetic model is solved using Kinetics Pre-Processor (KPP) version 2.2.3 (*77*), using the Rosenbrock solver and gfortran compiler.

#### Quantification of particle-bound organic radicals in SOA

Quantification of organic radicals in α-pinene–derived SOA is described in detail by Campbell *et al.* (*22*). In short, α-pinene SOA was produced by reacting known concentrations of α-pinene with O_3_ in flow tubes with four different reaction times. The SOA mixture was then bubbled through an impinger containing 40 ml of 50 μM spin trap 9-(1,1,3,3-tetramethylisoindolin-2-yloxyl-5-ethyn-yl)-10-(phenylethynyl)anthracene (BPEAnit) in dimethyl sulfoxide, which scavenges radicals to produce a stable fluorophore. The concentrations of radicals are then determined using fluorescence spectroscopy.

#### Cell cultures and cell exposures

Primary human bronchial epithelial cells were isolated from one human organ donor for all experiments described here. The lungs deemed not suitable for transplantation were approved for research use from the University of Kansas Institutional Review Board. Their use does not represent a human subject research as defined by Code of Federal Regulations (46.102).

Human bronchial epithelial cells were maintained in submerged two-dimensional culture in Bronchial Epithelial Cell Growth Medium (BEGM-LHC base media with supplements, Gibco, Thermo Fisher Scientific, Reinach, Switzerland). Cells were thereafter seeded onto porous 0.33-cm^2^ Transwell inserts (Corning International, Vitaris, Baar, Switzerland) in a chemically defined medium that induces terminal differentiation. Once confluent, the apical medium was removed, establishing ALI, and the epithelium allowed to differentiate over a period of 4 weeks. ALI cultures of reconstituted HBE were generated as previously described (*41*, *42*). Epithelial integrity, i.e., coordinated ciliary beating, mucus production, and maintenance of the ALI, was checked daily by light microscopy.

For online particle exposures, fully differentiated HBE were transferred to the NACIVT (Nano Aerosol Chamber for In Vitro toxicology) online particle deposition instrument under physiological conditions, i.e., 37°C, 5% CO_2_, and >85% relative humidity for 60 min. Details of the online particle exposure chamber are given by Jeannet *et al.* (*40*). Particle doses for online cell exposure ranged from 21 to 224 ng cm^−2^ (table S6) within the tracheobronchial dose range presented by Künzi *et al.* (*41*) (10 to 350 ng cm^−2^), which was calculated on the basis of an exposure range of 20 to 1000 μg m^−3^ PM mass concentration.

For the offline experiments, the same aerosol was collected on a filter and extracted in 1.5 ml of PBS or Hepes buffer, and 20 μl of the extracted aerosol was added to the fully differentiated HBE ALI cell cultures for 60 min.

Control ALI cell cultures were either exposed to particle-free air (p-free air) or were left untreated in the incubator [incubator control (inc. control)]. For p-free air exposure, we mounted a HEPA (high-efficiency particulate air) filter upstream of the NACIVT.

After exposure, cell cultures were incubated under the same conditions for 60 min before collecting apical wash samples and subsequently incubated for additional 23 hours followed by final sampling. At least three independent HBE cultures were used for each exposure.

### Cytotoxicity analysis

Induction of cell death was measured by the release of cytosolic LDH from damaged cells into the apical compartment. Apical washes were collected 4 and 24 hours postexposure and stored at 4°C until analysis using the colorimetric cytotoxicity detection kit^PLUS^ (Roche Diagnostics AG, Rotkreuz, Switzerland) according to the manufacturer’s instructions. Maximum releasable LDH was estimated in the supernatants of cells lysed with 100 μl of 1% Triton X solution for 10 min at 37°C. Cytotoxicity is presented as the percentage of maximal releasable LDH activity (absorbance).

### Quantitative real-time polymerase chain reaction

We screened 20 genes to evaluate alterations in signaling pathways related to oxidative stress using GeneGlobe arrays. Gene expression in HBE was examined by total RNA isolation followed by real-time quantitative polymerase chain reaction (PCR). Briefly, cells were lysed with RLT Buffer (RNeasy Mini Kit, Qiagen, Hombrechtikon, Switzerland) according to the manufacturer’s protocol. RLT buffer was added with β-mercaptoethanol, and samples were stored at −80°C until further processing. Isolated RNA (500 ng) was reverse transcribed into cDNA using QuantiTect Reverse Transcription Kit (Qiagen) following the manufacturer’s recommendations. Real-time PCR was performed in a reaction volume of 25 μl using the QuantiTect SYBR Green PCR kit (Qiagen) and the QuantiTect Primer Assays (Qiagen), amplifying a total of 25 ng of cDNA of each sample. Real-time PCR was performed using the Applied Biosystems 7900HT-Fast Real-Time PCR System with a 15-min initial activation step at 95°C and 40 cycles with 15-s denaturation at 94°C, 30-s annealing at 55°C, and 30-s extension at 72°C. Subsequently, a melting curve was performed to exclude primer-dimer artifacts and to ensure reaction specificity. Data were normalized to hypoxanthine-guanine phosphoribosyl transferase using the ΔΔ*C*_t_ method (*78*). Biological replicates (*n* = 3) were analyzed three times using Applied Biosystems SDS version 2.4.

### Proteome profile

The inflammatory response was evaluated by measuring the release of 102 cytokines and chemokines from cells using the Proteome Profiler Human XL Cytokine Array (ARY022, R&D Systems, Minneapolis, MN). The kit contained all the reagents for the assay and was performed as per the manufacturer’s instructions. This cytokine and chemokine antibody array was used to determine the effects of α-pinene SOA and naphthalene SOA particle exposure on cytokine and chemokine release by HBE. The assay required 1500 μl of pooled cell culture basal media (500 μl of basal media for each cell culture, *n* = 3). Membranes were subjected to an ultrasensitive chromogenic 3,3′,5,5′-tetramethylbenzidine membrane substrate (Thermo Fisher Scientific, Waltham, MA) to reveal sample-antibody complexes labeled with streptavidin-HRP. Photographs of the blots were taken after exposure to the substrate.

### Statistical analysis of biological results

Statistical analyses were performed using the commercial software GraphPad Prism 7.04 (GraphPad Software Inc., San Diego, US). For cytotoxicity, the arithmetic mean values of each experiment were compared to the mean value of the untreated control by a one-way analysis of variance (ANOVA) followed by Dunnett’s *t* test to compare the treated group to the control or the Bonferroni test for multiple comparisons.

Statistical analysis for gene expression and proteome was determined with the commercial software GraphPad Prism 7.04 for Windows (GraphPad Software Inc., La Jolla, CA). Nonmatching one-way ANOVA with Turkey’s multiple comparison tests was used for the statistical comparison to untreated control. Differences that show a fold change of at least 1.5 and satisfy *P* < 0.05 after the adjustment for multiple testing were considered significant.

PCA was done using SIMCA 17 (Sartorius, Germany). For genes, a total of 20 (60 samples of triplicate exposures analyzed) non-normalized gene expression values were considered, while for proteins, 102 non-normalized values were used. Model performance was evaluated using *R*^2^ values as a measure of proportion of variance explained by the model and by the *Q*^2^ value, which estimates the predictive power of the model through sevenfold cross-validation using randomly selected test/train subsets taken from the whole dataset. Hotelling’s *T*^2^ statistics, represented by the gray ellipse in Fig. 4 (B and D), was used to identify potential outliers in the dataset using the multivariate probability distribution.
