# Supplementary material for: Short-lived reactive components substantially contribute to particulate matter oxidative potential
Source: Sci Adv. 2025 Mar 19;11(12):eadp8100. doi: 10.1126/sciadv.adp8100 (PMC11922065; doi:10.1126/sciadv.adp8100)
Supplement: Supplementary file 1 — Supplementary Text Figs. S1 to S7 Tables S1 to S4 [file sciadv.adp8100_sm.pdf]

Science Advances
AAAS

Supplementary Materials for

Short-lived reactive components substantially contribute to particulate matter oxidative potential

Steven J. Campbell *et al.*

Corresponding author: Steven J. Campbell, steven.campbell@imperial.ac.uk;
Markus Kalberer, markus.kalberer@unibas.ch

Sci. Adv. **11**, eadp8100 (2025)
DOI: 10.1126/sciadv.adp8100

This PDF file includes:

Supplementary Text
Figs. S1 to S7
Tables S1 to S4

Supplementary Text

Overall setup of the Online Laboratory Experiments to Characterize OP, ROS and Biological Effects

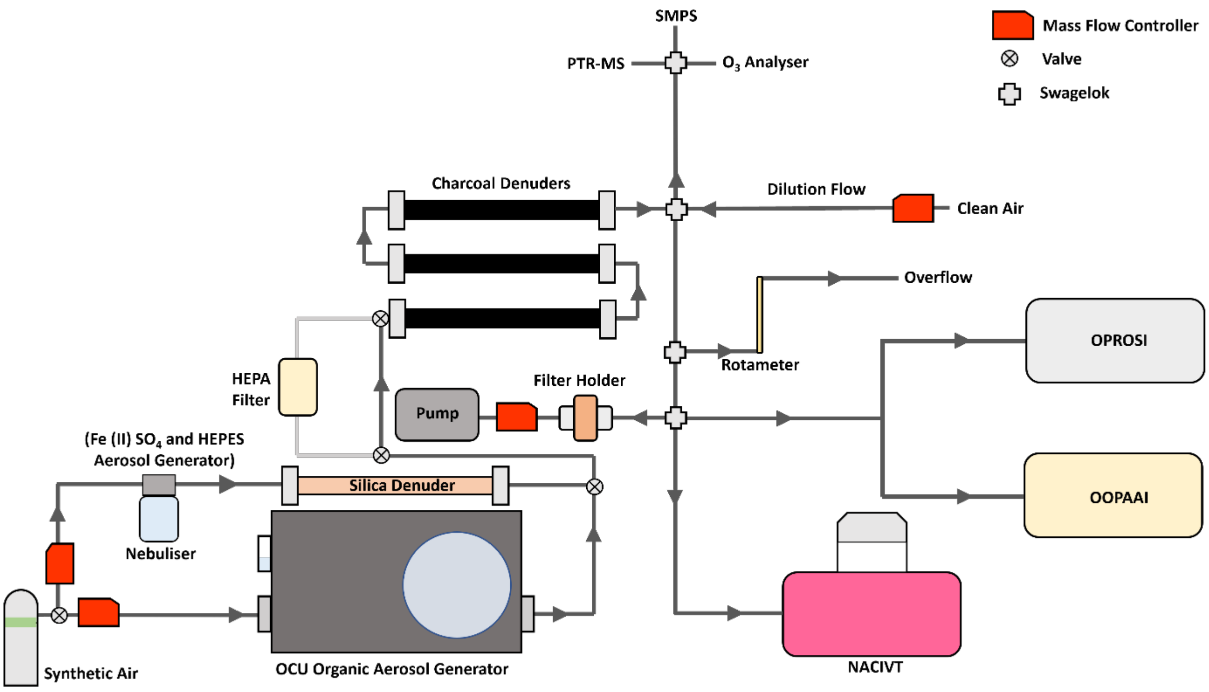

Fig. S1.

Schematic representation of the experimental setup used in this study to generate SOA, Fe(II)SO_4 and HEPES particles for online and offline cell exposure experiment, as well as for online and offline ROS and OP quantification. Particle size distribution, O_3 concentration and volatile organic compounds were monitored as well.

Additional Aerosol ROS and OP Data

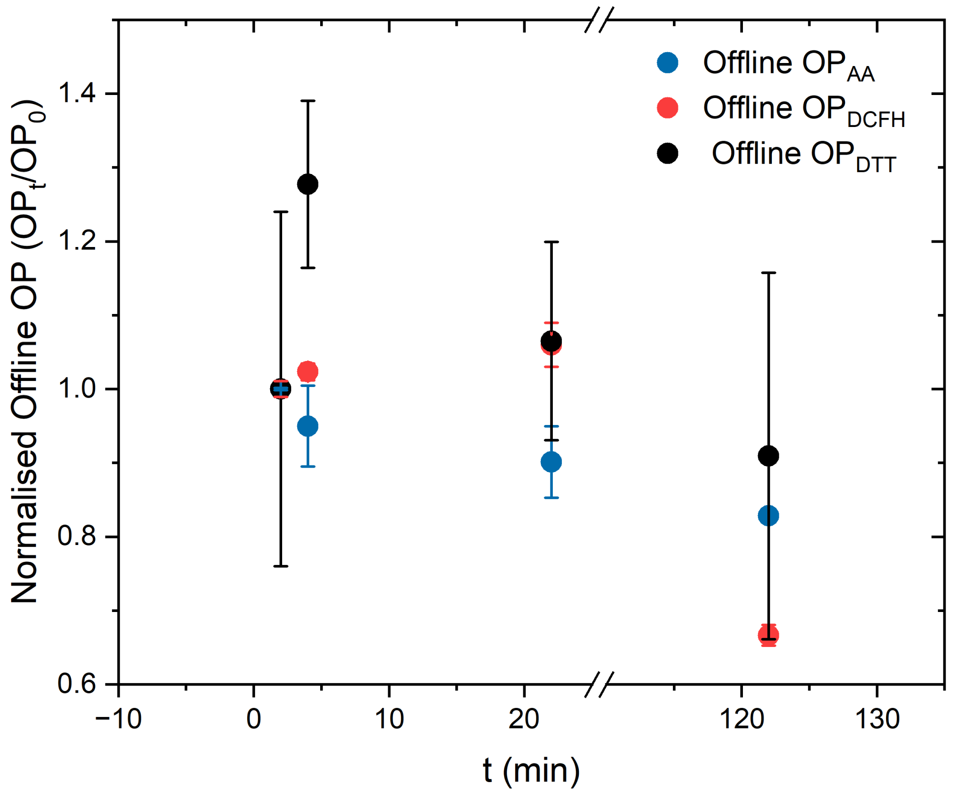

Fig. S2.

The same data as presented in Fig. 1A but showing only the decay of OP and ROS for the first 120 minutes. OP and ROS concentrations of samples analysed after 2 min and 5 min, respectively, vary but are within errors. The relatively large errors at these shortest analysis times are due to the very fast particle collection, samples handling and extraction times of only a few minutes, causing more uncertainties. In the data point where no error bars are visible (OP$_{DCFH}$), errors are within the size of the respective data point.

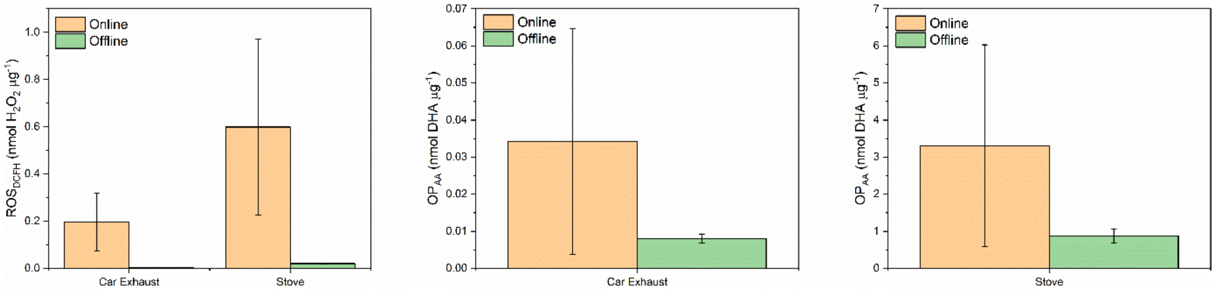

Fig. S3.

Comparison of online (orange) and offline (green) measurements of OP_{DCFH} and OP_{AA} of aged car exhaust and aged wood burning particles; (A) ROS_{DCFH} for both car and wood stove aerosol, (B) OP_{AA} of car exhaust aerosol and (C) OP_{AA} of wood stove aerosol. Error bars for online measurements represent the variability in online signal over time due to different experimental conditions (e.g., burning phase of wood stove emissions) and not measurement uncertainty. Error bars for offline measurements represent the standard deviation observed over three experimental repeats.

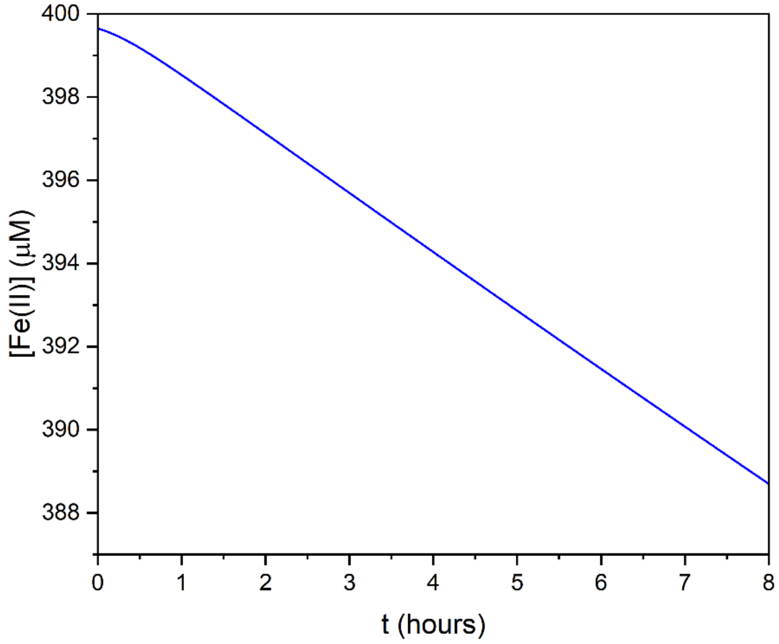

Fig. S4.
Kinetic model for oxidation of Fe (II) to Fe(III) at pH 5, replicating conditions in the nebulizer during our experiments. < 3% Fe (II) is converted to Fe (III) via aqueous oxidation over an 8-hour period. Fe (II) oxidation was modelled using the kinetic model extensively described in Shen et al. *(32)* and Campbell et al. *(37)*.

Table S1.

Percentage of short-lived ROS_{DCFH}, OP_{AA}, OP_{OH}, OP_{DTT} and OP_{BPEA} lost in offline analyses (ratio of online to offline quantification). Time delay between aerosol sampling and offline analysis is indicated in parenthesis where available.

Particle type	ROS_{DCFH}	OP_{AA}	OP_{OH}	OP_{BPEA}	References	Notes
β-pinene-SOA (1h (1), 6 months (2))	93 - 97	67	--	--	(1) This study<br>(2) Zhang et al. (2022) *(24)*	$PM_{2.5}$
Limonene-SOA (20min)	60 - 75	--	--	--	Gallimore et al. (2017) *(79)*	$PM_{2.5}$
Naphthalene-SOA (1h (1), 6 months (2))	92 - 94	95	--	--	(1) This study<br>(2) Zhang et al. (2022) *(24)*	$PM_{2.5}$
Oxidized oleic acid (24h)	> 90	--	--	--	Fuller et al. (2014) *(23)*	$PM_{2.5}$
Fe(II)SO_4 (1 h)	--	~ 0	--	--	This study	$PM_{2.5}$
Aged car exhaust (24 h)	79	76		--	This study	$PM_{2.5}$
Aged wood burning (24 h)	97	74	--	--	This study	$PM_{2.5}$
Ambient London (6 months)	93 – 99	--	--	--	This study	$PM_{2.5}$
Ambient Padua (6 months)	96 – 99	--	--	--	This study	$PM_{2.5}$
Ambient Beijing (6 months)	99	--	--	--	This study	$PM_{2.5}$
Ambient Los Angeles (2 weeks)	--	--	80	--	This study	PM_1, $PM_{2.5}$, TSP
Ambient Los Angeles (UCLA)	--	--	75 (TSP) 95 ($PM_{2.5}$)		Taghvaee et al. (2023) *(49)*	$PM_{2.5}$ and total suspended particulates (TSP)
Ambient Switzerland (up to 4 months)	60 ± 20	--	--	--	Zhou et al. (2018) *(50)*	$PM_{2.5}$
Ambient Georgia, Atlanta (3 hr)	7	--	--	--	King et al. (2013) *(28)*	($PM_{2.5}$) Mist chamber used to collect particles prior to online analysis.
Ambient Heshan, China (--)	--	--	--	71%	Brown et al. (2020) *(52)*	$PM_{2.5}$, short lived fraction estimated using modelling techniques.

Additional Cell Exposure Results

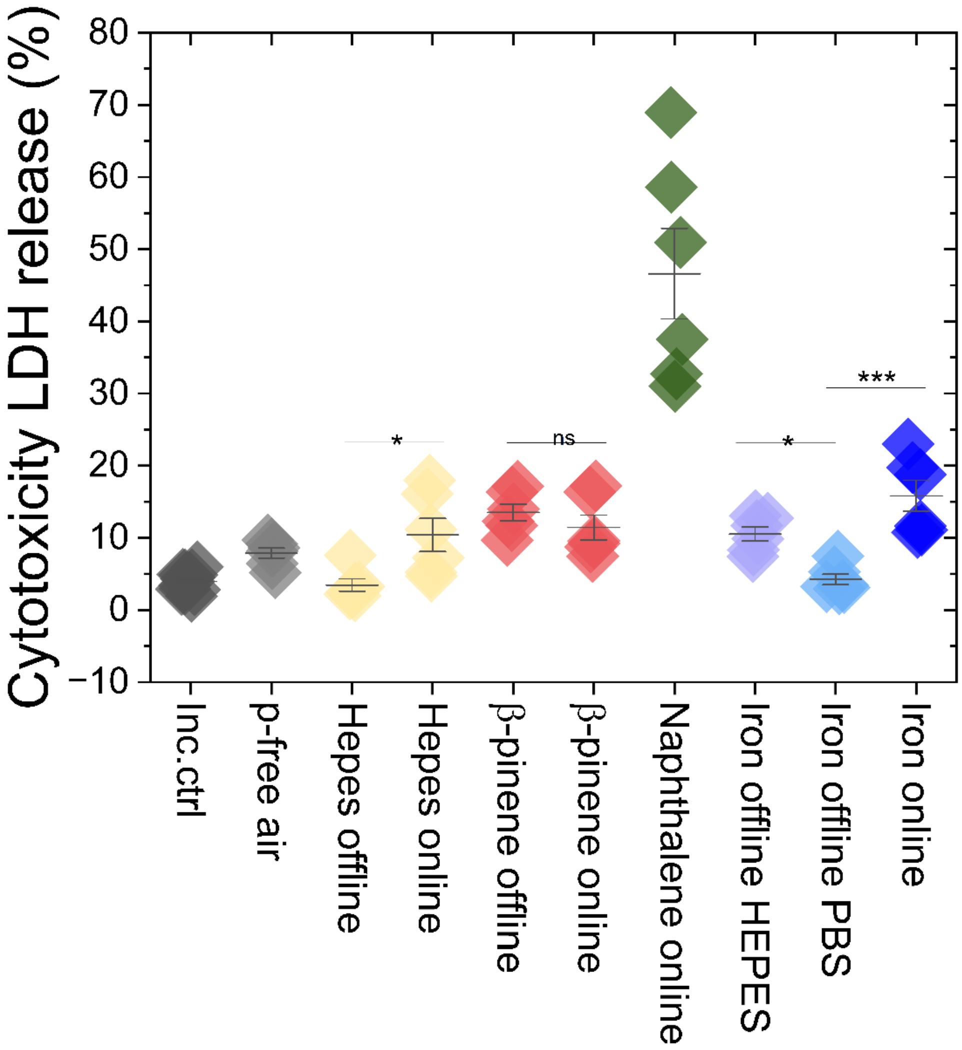

Fig. S5.

Cytotoxicity (measured as LDH release in % of the maximum amount of released LDH 24 hours after particle exposure) of a range of particle types and for online deposition and offline exposure.

The by far largest cytotoxicity is observed for anthropogenic naphthalene-SOA, followed by iron particles, biogenic β-pinene-SOA and HEPES-buffer particles (Fig. S5), supporting the gene activation results (Fig. 4).

Incubator control and particle-free air control exposure have the same low LDH values, demonstrating that the treatment of the cells in the NACIVT online cell exposure chamber does not affect biological functions of the cells related to normal turnover. Up to 10% LDH is considered a normal LDH release level for the primary cells used in our study. LDH measurements 4 hours after the particle exposure show < 1% values of the 24-hour data, illustrating that the particle toxicity effect is a rather slow process. The generally low LDH levels illustrate that the

doses applied here (see Table S2) were realistic as they did not cause dramatic toxicity (i.e., necrosis), which is usually only assumed to be significant when LDH release > 15% is observed.

Anthropogenic naphthalene-SOA particles are the only particle types where LDH release is substantially above this threshold, indicating the increased toxicity of this particle type at particle doses of 224 ng/cm^2. Biogenic β-pinene-SOA show much lower LDH levels at comparable doses (i.e., 188 ng/cm^2). However, if the β-pinene-SOA dose was increased by almost two orders of magnitude (> 10 $\mu g/cm^2$), then LDH release levels similar to naphthalene-SOA at 224 ng/cm^2 were observed, as illustrated in the dose-response curve shown in Fig. S6.

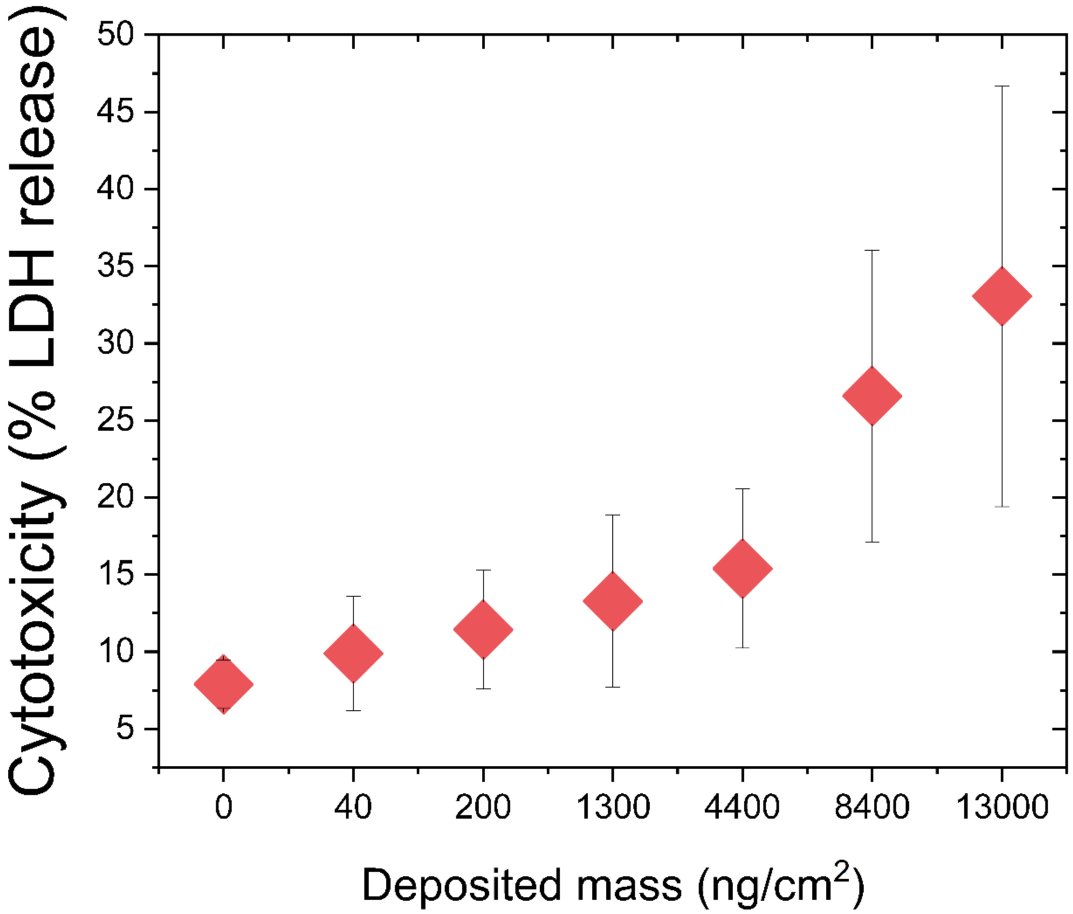

Fig. S6.
Dose response of cytotoxicity for β-pinene-SOA quantified with the online particle deposition set up (Fig. S1).

Cell Exposure to $Fe(II)SO_4$ particles (Fig. S7)

$Fe(II)SO_4$ particles are water soluble and Fe(II) is known to generate ROS through Fenton-like chemistry in aqueous media, while Fe(II) will be chemically stable during offline handling (Fig. S4). Therefore, a strong OP-related biological response upon HBE exposure to Fe(II) is expected but there should be no OP-related *differences* in the biological response comparing online/offline exposures.

Only a slight enhancement of gene expression was observed when cells were directly exposed to $Fe(II)SO_4$ particles using online methods, compared to offline exposure (Fig. 4A, 4B, S7). This is expected, because the chemical composition of iron sulphate particles does not change between offline and online exposure methods. However, for protein expression, very pronounced differences were observed between online and offline deposition of $Fe(II)SO_4$ particles (Fig. S7). This is likely explained by the presence of a buffer (PBS and HEPES buffer were tested) needed in the particle extracts for offline deposition (adding particle extracts without buffer to cells would cause strong cell responses). Buffers are complexing Fe(II) ions in aqueous solution causing possibly the significant decrease protein expression, close to levels of the control samples (Fig. S7C, S7D) and thus masking its toxicity. PBS is known to complex Fe while HEPES is often considered as one of the buffers least interfering with metal reactivity but the data presented here illustrate that HEPES also lowers the toxicity of iron considerably.

This illustrates that during offline particle exposure, unexpected and undesired chemical changes of the particle reactivity can occur, which are challenging or impossible to identify if no concurrent online particle deposition experiments are performed.

Cytotoxicity (LDH) results support the protein expression differences for online and offline exposures. Online iron deposition shows an enhanced LDH release, whereas after offline exposure, the LDH release was close to incubator and particle-fee air controls.

For a realistic assessment of particle-cell interactions, a buffer-free exposure is therefore required, which can only be achieved with solvent- and buffer-free methods, i.e., with online particle deposition.

Cell Exposure to HEPES-buffer particles (Fig.7)

HEPES-buffer particles serve as negative controls in the particle exposure experiments summarised in Fig. 4, Fig. S5 and Fig.S7. HEPES buffer is used during standard cell cultivation protocols, and therefore considered non-toxic for online as well as offline deposition *(43)*. Exposure of HBE to HEPES-buffer particles should therefore not promote chemistry-driven toxicity, and was performed to explore if mechanical deposition of particles onto cells during online exposure induces any toxicity responses. On all three levels (i.e., gene activation, protein expression and cytotoxicity), HEPES-buffer particles did not induce increased responses compared to controls, and there were no significant differences were observed between online and offline HEPES particle exposures. This demonstrates that differences observed in offline and online exposure to SOA is likely due to changes in chemistry (e.g., ROS and OP), and not induced by the deposition process of particles during online exposure.

Table S2.

Deposited particle masses during biological experiments. For offline exposures, particles were generated in the same was as for online analysis, but were collected onto a filter as described in Fig S1. The same particle concentrations were gerenated under the same conditions for online analaysis and offline collection using the organic coating unit (OCU) to ensure comparable composiotion of SOA for online/offline comparison.

**Aerosol type**	**Mass (ng) deposited per cell culture insert**	**Particle concentration during online expsures (μg/m^3)**
particle-free air	0.0	0.1
HEPES offline	108.6	n.a.
HEPES online	115.0	25.5
β-pinene offline	181.4	n.a.
β-pinene online	188.1	41.6
Naphthalene online	224.6	49.7
Iron offline HEPES	18.6	n.a.
Iron offline PBS	18.6	n.a.
Iron online	21.2	4.7

Table S3.
Abbreviations of Genes

Abbreviation	Full Name
IL-6	Interleukin-6
IL-8	Interleukin-8
S100A9	S100 calcium-binding protein A9
DUOX1	Dual oxidase 1
DUOX2	Dual oxidase 2
NOX4	NADPH oxidase 4
CFLAR	CASP8 and FADD-like apoptosis regulator
SOD1	Superoxide dismutase 1
SOD2	Superoxide dismutase 2 (mitochondrial)
SOD3	Superoxide dismutase 3
NFE2L2	Nuclear factor erythroid 2-related factor 2
GPX1	Glutathione peroxidase 1
HMOX1	Heme oxygenase 1
NQO1	NAD(P)H quinone dehydrogenase 1
PRDX2	Peroxiredoxin-2
SRXN1	Sulfiredoxin-1
GSTA1	Glutathione S-transferase alpha 1
SCARA3	Scavenger receptor class A member 3 (SCARA3)

Table S4.
Abbreviations of Proteins

**Abbreviation**	**Full Name**
C5/C5a	Complement Component 5 / Complement C5a
IL-23	Interleukin-23
IL-27	Interleukin-27
IL-34	Interleukin-34
IP-10	Interferon Gamma-Induced Protein 10
CD14	Cluster of Differentiation 14
CD30	Cluster of Differentiation 30
IL-1alpha	Interleukin-1 alpha
IL-32	Interleukin-32
GRO-alpha	Growth-Regulated Oncogene Alpha
IL-4	Interleukin-4
IL-8	Interleukin-8
IL-13	Interleukin-13
IL-33	Interleukin-33
IL-6	Interleukin-6
IL-11	Interleukin-11
IL-19	Interleukin-19
TSP-1	Thrombospondin-1
ADIPOQ	Adiponectin
IL-10	Interleukin-10
